# Diffuse Large B-cell Lymphoma Presenting as a Primary Pleural Mass: A Case Report and Literature Review

**DOI:** 10.7759/cureus.22765

**Published:** 2022-03-02

**Authors:** Gia Thinh D Truong, Zachary A Creech, Kurt V Shaffer, Matthew Merrill

**Affiliations:** 1 Internal Medicine, Creighton University School of Medicine, Phoenix, USA; 2 Internal Medicine, Creighton University School of Medicine, St. Joseph's Hospital and Medical Center, Phoenix, USA

**Keywords:** complex pleural effusion, delayed diagnosis, pleural cavity, diffuse large b cell lymphoma (dlbcl), clinical case report

## Abstract

Primary pleural lymphoma is a rare type of lymphoma that accounts for only ​​0.3% of all non-Hodgkin's lymphomas. The rarity and nonspecific clinical presentation of primary pleural lymphomas pose a diagnostic challenge for clinicians. We present an atypical case of primary pleural lymphoma in an elderly patient without any associated pleuro-pulmonary disease, immunosuppression, or history of lymphoma. To our knowledge, this is one of the first described cases of a primary pleural lymphoma with such a presentation.

## Introduction

Primary pleural lymphoma is a rare type of lymphoma. Secondary spread of lymphoma to distinct anatomical regions, including the pleura, is a well-documented phenomenon [1]. However, the pleura is an uncommon site of primary tumorigenesis, accounting for only 0.3% of all non-Hodgkin's lymphomas [2]. Current classifications divide primary pleural lymphomas into two categories: primary effusion lymphoma (PEL) and diffuse large B-cell lymphoma (DLBCL) associated with chronic inflammation (DLBCL-CI) [3]. Notably, these entities have been associated with immunosuppression, chronic pyothorax, and prior trauma or pathological involvement of the pleura [3]. We present a case of primary pleural DLBCL in a patient without a prior history of known risk factors and provide a short review of the current literature for these tumors.

## Case presentation

An 82-year-old female presented with a one-day history of sudden-onset, sharp, pleuritic chest pain radiating to the left arm, shoulder, and upper back with associated mild exertional dyspnea. She denied any history of fever, night sweats, weight loss, or cough. Her past medical history was notable for a prior squamous cell carcinoma of the nose that was excised eight years prior without documented recurrence or spread. She denied any history of HIV infection, occupational exposure, or smoking.

On initial examination, the patient was alert and displayed no signs of distress. There were no significant physical exam findings. There was no evidence of peripheral lymphadenopathy or hepatosplenomegaly. Her blood pressure was 174/80 mmHg, heart rate was 67 bpm, respiratory rate was 18/min, oxygen saturation (SpO2) was 99% while breathing room air, and her temperature was 36.6°C. Laboratory studies were notable for a white blood cell count of 12,500 cells/µL, with a neutrophilic predominance, erythrocyte sedimentation rate of 33 mm/hour, and a C-reactive protein level of 15.6 mg/L.

**Figure 1:**
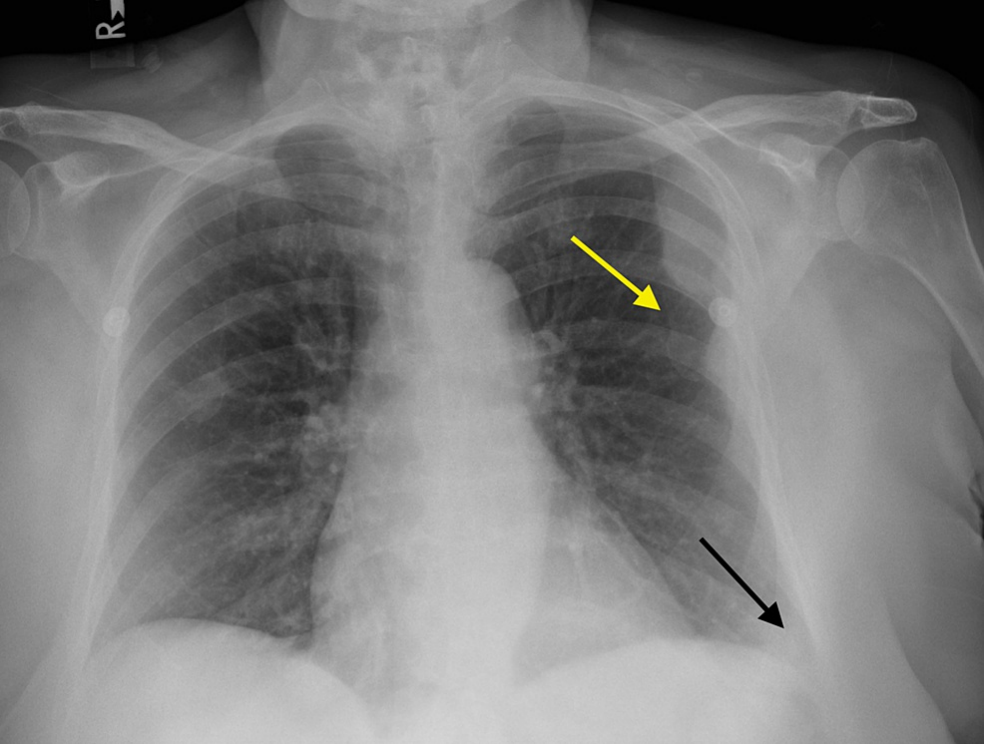
Chest X-ray of the patient. Chest X-ray showing a lateral, left-upper-lung-zone, convex opacity measuring approximately 7.0 x 3.3 cm (yellow arrow). A trace left-sided pleural effusion (black arrow) is also present.

Chest radiography demonstrated an opacity measuring approximately 7.0 x 3.3 cm in the left posterolateral chest cavity and a small left pleural effusion (Figure 1). A CT of the chest revealed a lobulated extra-pleural mass with involvement of the posterolateral chest wall and fifth rib. Notably, no other mediastinal or lung parenchymal masses were visualized. At that time, a decision was made to pursue a CT-guided biopsy of the mass and to delay any intervention on the effusion until further information was available. Histological staining of several core-needle specimens from the mass revealed a population of atypical, medium-to-large lymphoid cells with irregular nuclear contours and multilobate nuclei. Immunostaining was positive for CD20, CD79a, B-cell lymphoma 6 (Bcl-6), multiple myeloma oncogene 1 (MUM-1), B-cell lymphoma 2 (Bcl-2), and CD23 and negative for CD5 and CD10. Flow-cytometry revealed a kappa chain restricted population of B-cells. Altogether, these findings were thought to be consistent with primary pleural diffuse large B-cell lymphoma.

This patient was a primary resident of another state, and therefore, elected to leave our institution after undergoing a biopsy. As a result, she was lost to follow-up after being informed of the diagnosis and the remaining course of her illness is unknown to us at this time.

## Discussion

DLBCL is the most common type of non-Hodgkin's lymphoma (NHL). DLBCL is more common in the elderly population, but can arise in young adults and rarely in children [4]. It is known to present in an array of nodal and extranodal locations, including the mediastinum, nasal sinuses, salivary glands, skin, and most commonly, the gastrointestinal tract [4-6].

DLBCL predominantly arises as an NHL, but may also arise as a Hodgkin’s lymphoma [5,7]. In general, secondary involvement of other anatomical areas, including the pleura, is common in lymphoma [1]. However, the pleura is an uncommon site of primary oncogenesis, accounting for only 0.3% of all NHLs [2]. Similarly, the occurrence of primary pulmonary B-cell lymphoma (PP-BCL) is rare, accounting for less than 1% of NHLs. This patient’s presentation of a primary pleural mass involving the posterolateral chest wall makes this a rare case [8].

The majority of cases of primary pleural lymphoma (PPL) in the literature are characterized as either PEL or pyothorax-associated lymphoma (PAL) [7]. Currently, the WHO Classification of Tumors 2015 lists two types of PPLs, including PEL associated with human herpesvirus 8 (HHV-8) and DLBCL-CL associated with Epstein-Barr virus (EBV) infection [3].

PPLs have a diverse and often nonspecific clinical presentation [9]. Malignant masses arising from the pleura are rare and difficult to identify due to their proximity to and possible involvement of other anatomical sites. Additionally, the limited available literature and non-specific presenting symptoms, such as dyspnea, cough, hemoptysis, and pleuritic pain, add to the difficulty of diagnosis. Prior reports have documented presenting symptoms of pulmonary lymphomas ranging from cough, dyspnea, chest pain, and even hemoptysis, but oftentimes the patient is asymptomatic and the lesion is found incidentally on imaging [10]. Naturally, much of this symptomatology is consistent with that of lymphomas elsewhere in the body or with other pleural pathology. Patients with early-stage lymphoma are generally asymptomatic, with only 30-45% presenting with nonspecific “B symptoms” that include fever, weight loss, and night sweats [10,11]. The clinical presentation of PPL can therefore lead to a delayed diagnosis or misdiagnosis.

The development of pleural effusion may also increase the risk of misdiagnosis. Our patient’s primary presenting symptom was pleuritic pain, one of the most common presenting symptoms of both PAL and PEL, and she was also found to have a small pleural effusion [12]. Pleural effusions have been reported in roughly 25% of patients with NHL and are often misdiagnosed as either tuberculosis or tuberculous pleurisy [2,11,13]. Tuberculosis and tuberculous pleurisy are common misdiagnoses due to shared clinical characteristics with PPL including elevated adenosine deaminase levels, B symptoms, and fatigue [2,11]. Other reported reasons for misdiagnosis of PPL included patient hesitancy for invasive procedures and difficulty of biopsy, which is required for diagnostic confirmation [2]. The vast symptomatology of these lesions highlights the importance of a broad differential and comprehensive medical review to reach an accurate and timely diagnosis.

Reported cases of PPL in the literature have been associated with immunosuppression, chronic pleural inflammation, or a prior history of pathological involvement of the pleura. Interestingly, most of the reported cases were from Japan [7]. Iuchi et al. reviewed 37 cases of PPL in Japanese hospitals and found that all patients included in the study had a long-standing history of pyothorax resulting from tuberculous pleuritis or the treatment of pulmonary tuberculosis [14]. This association is in line with the proposed pathogenesis of PPL, which attributes a significant role to the presence of chronic inflammatory states. It is suggested that the chronic stimulation of cytokines establishes an immunosuppressive environment, which is crucial for the growth of the lymphoma [12]. It has also been proposed that autoimmune diseases and infections such as EBV are strongly associated with the development of PAL [12]. A study in 2004 reported a significant increase in the risk of lymphoma in patients with severe rheumatoid arthritis [15]. Additionally, trauma to the thorax has also been shown to be associated with PAL, highlighting the potential role of post-traumatic inflammation in tumor development [16]. However, Sun et al. reported a case of PPL that was negative for chronic pyothorax, or any indications of HIV or mycobacterium infection, suggesting other pathological mechanisms for the development of these malignancies [7]. Reported cases of PPLs in current literature are presented in Table 1.

**Table 1:** Summary of features of primary pleural lymphoma cases reported in the literature. The features of other reported cases of primary pleural lymphoma in the literature are presented in this table. DLBCL, diffuse large B-cell lymphoma.

Study	Age and gender	Primary site	Other structures involved	Cancer type	Treatment; outcome
Yang et al. [2]	49, male	Left pleura	Chest wall, presence of left pleural effusion	DLBCL	Deceased
Rabadão et al. [5]	81, female	Right superior lobe of pleura	Long-standing pleural effusion, heart failure	DLBCL	Cyclophosphamide and prednisolone; remission
Sun et al. [7]	73, male	Left pleura	Presence of left pleural effusion	DLBCL	Cyclophosphamide, pirarubicin, vincristine, and prednisolone (CHOP) chemotherapy; remission
Ru et al. [9]	74, female	Right pleural cavity	Anemia, right-sided pleural effusion	Small B-cell lymphocytic lymphoma (SLL)	Surgery; remission
Sun et al. [7]	Reported cases of 12 patients	Pleura	Not available	DLBCL	Treatment included a combination of chemotherapy, radiation therapy, and surgical resection
Vega et al. [13]	Reported cases of five patients	Pleura	Not available	DLBCL	Not available

A biopsy is a primary method to address diagnostically challenging pleural effusions and masses, often requiring a combination of histopathological and immunohistochemical analyses [17]. DLBCLs can be classified into the germinal center and non-germinal center lymphomas based on their immunostaining with CD10, BLC6, and MUM-1 [18]. Our patient’s immunohistology results were negative for CD10, but positive for BCL6 and MUM-1, likely suggesting a non-germinal-center lymphoma. In addition to immunohistochemical markers, the MIB-1 proliferation index can also be used to identify indolent, aggressive, and highly aggressive lymphomas [19]. Our patient had a MIB-1 proliferative index of 30%, indicating an indolent B-cell lymphoma. A fluorodeoxyglucose positron emission tomography/computed tomography (FDG PET/CT) scan would be an effective method to confirm the diagnosis and obtain more prognostic information. An FDG PET/CT scan has been shown to have a sensitivity of 95-97% and a specificity of up to 95% in identifying pleural malignancies [20]. Additionally, FDG PET/CT imaging can be used to distinguish between solid pleural disease from pleural effusion [1]. This information is useful in planning and assessing therapy options; however, our patient was lost to follow-up.

## Conclusions

This case presentation of a PPL in an elderly patient without any associated pleuro-pulmonary disease or history of lymphoma is extremely rare. Previous reports suggest chronic inflammation, thoracic trauma, long-term pyothorax, and injury as possible factors contributing to the development of lymphoma of the chest wall or pleura, none of which were present in this patient. To our knowledge, this is one of the first described cases of a PPL with such a presentation and may indicate a need to include PPL in the differential of unidentified pleural masses in the absence of known risk factors. By presenting this rare case, we hope to add to the existing literature and improve the management and treatment of future patients.
